# Pulmonary alveolar proteinosis developing during steroid treatment in a patient with organizing pneumonia in association with atypical chronic myeloid leukemia

**DOI:** 10.1002/ccr3.2014

**Published:** 2019-01-31

**Authors:** Chiaki Hosoda, Keisuke Saito, Shota Fujimoto, Yumie Yamanaka, Naoaki Watanabe, Hanae Miyagawa, Yusuke Kurita, Yoshitaka Seki, Akira Kinoshita, Yasuhiko Endo, Kazuyoshi Kuwano

**Affiliations:** ^1^ Department of Internal Medicine, Division of Respiratory Medicine The Jikei University Daisan Hospital Tokyo Japan; ^2^ Department of Pathology The Jikei University School of Medicine Tokyo Japan; ^3^ Department of Internal Medicine, Division of Respiratory Medicine The Jikei University School of Medicine Tokyo Japan

**Keywords:** chronic myeloid leukemia, corticosteroid therapy, organizing pneumonia, pulmonary alveolar proteinosis

## Abstract

Organizing pneumonia (OP) and pulmonary alveolar proteinosis (PAP) are rare complications in patients with hematologic disorders. We herein report a case of PAP that developed during steroid treatment for OP in a patient with atypical chronic myeloid leukemia. Physicians should pay close attention to these complications in patients with hematologic malignancies.

## 1 INTRODUCTION

Pulmonary alveolar proteinosis (PAP) is an uncommon disease characterized by the accumulation of surfactant proteins and phospholipids within the alveolar spaces.^1^, ^2^ Acquired PAP is divided into two forms based on its clinical features: idiopathic PAP and secondary PAP (sPAP). sPAP most commonly occurs in association with hematologic malignancies.^3^ Organizing pneumonia (OP) has also been reported as a rare complication in patients with hematologic malignancies.^4^ To our knowledge, there have been no reports of the development of both OP and PAP in the same patient.

We herein report a case of hematologic malignancy‐associated PAP with a poor outcome that developed during steroid treatment for OP.

## 2 CASE REPORT

**Figure 1:**
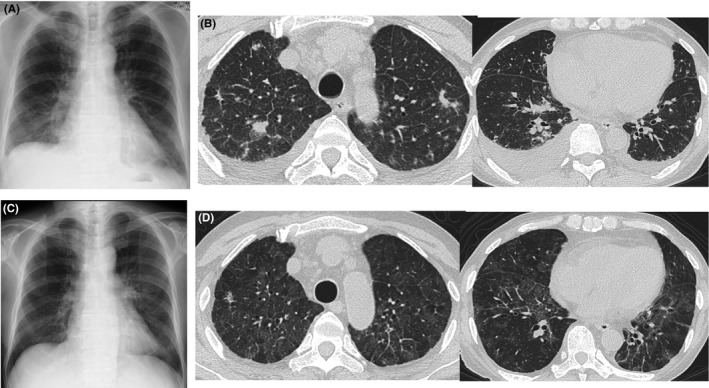
Radiological findings. A and B, Chest X‐ray and computed tomography (CT) at the initial consultation revealed scattered small nodular shadows, patchy consolidation, and bilateral pleural effusion. C and D, Six months after the diagnosis of organizing pneumonia, chest X‐ray and CT showed diffuse ground‐glass opacity

The patient was a Japanese 72‐year‐old man, who had been diagnosed with atypical chronic myeloid leukemia (aCML) in 2014. He was an ex‐smoker who did not regularly consume alcohol. In September 2015, treatment with oral cytarabine ocfosfate hydrate was initiated. After four cycles, he developed pneumonia, and treatment was terminated in February 2016. In April 2016, although he had no complaints, his serum C‐reactive protein level was found to have re‐increased to 4.6 mg/dL, and a chest X‐ray and high‐resolution computed tomography (HRCT) revealed scattered small nodular shadows and patchy consolidation (Figure 1A,B). The radiological findings did not improve despite the administration of antibiotics and antifungal drugs.

**Figure 2:**
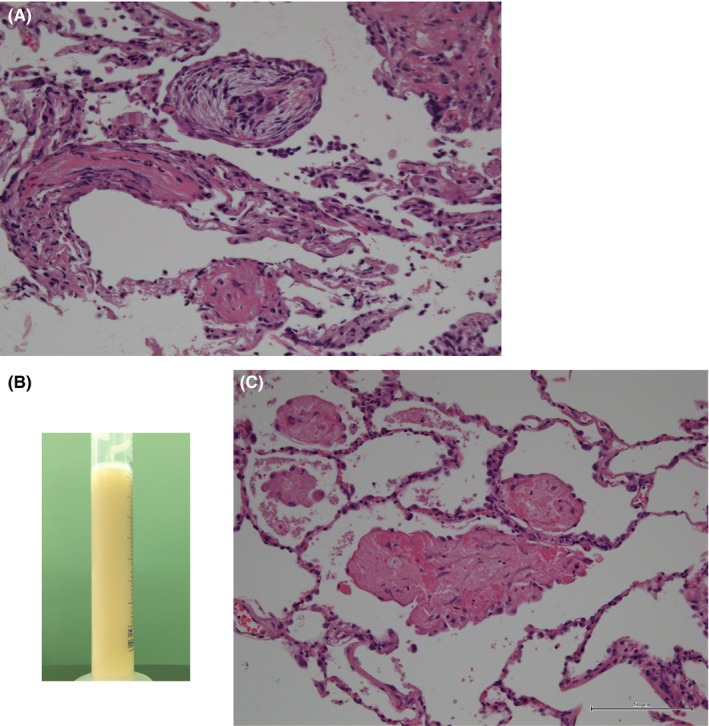
A, The pathological examination of a transbronchial lung biopsy specimen obtained in May 2016 revealed a Masson body and airspace organization (×400, Hematoxylin and Eosin (HE) staining). B, The bronchoalveolar lavage fluid showed a light milky appearance. C, The pathological examination of a transbronchial lung biopsy specimen showed eosinophilic dense homogenous materials filling the alveolar septa (×400, HE staining)

**Figure 3:**
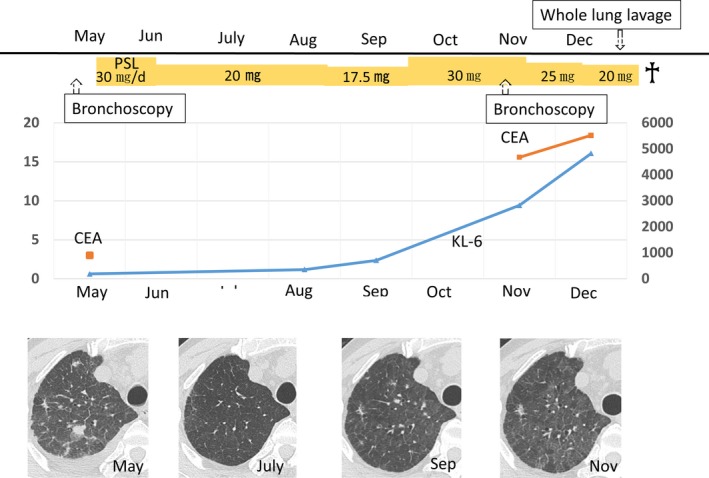
The clinical course of the patient's laboratory data and therapies. CEA, carcinoembryonic antigen; KL‐6, Krebs von den Lugen‐6; PSL, prednisolone

We performed bronchoscopy in May 2016. Bronchoalveolar lavage performed in the right upper lobe recovered 90 mL of 150 mL (60%) with 1.3 × 10^5^/mL cells (neutrophils: 26%, lymphocytes: 36%, eosinophils: 1%, and macrophages: 37%). The histological examination of a specimen obtained from the right upper lobe via transbronchial lung biopsy revealed findings consistent with OP (Figure 2A). On immunofluorescence testing, the patient's antinuclear antibody titer was <40, and no other autoantibodies, including anti‐SS‐A, anti‐aminoacyl tRNA synthetase antibody, rheumatoid factor, and anti‐cyclic citrullinated peptide antibody, were detected. We diagnosed the patient with secondary OP associated with aCML. Treatment with prednisolone (30 mg, daily) was initiated, which resulted in the improvement of the laboratory and radiological findings, and the dose of prednisolone was then gradually tapered (Figure 3). In September 2016, the patient developed general fatigue while under treatment with prednisolone (17.5 mg, daily). Chest CT revealed diffuse ground‐glass opacities (GGOs). We considered the possibility of a recurrence of OP, and therefore increased the dose of prednisolone to 30 mg, daily; however, the patient's condition did not improve.

The patient was admitted to our hospital due to dyspnea on effort in November 2016. On admission, a physical examination revealed the following findings: respiratory rate, 15 breaths per minute; heart rate, 80 beats per minute; blood pressure, 106/60 mm Hg; and body temperature, 37.3°C. Chest auscultation revealed no abnormalities.

The laboratory tests performed on admission included an arterial blood gas analysis under ambient air, which showed the following findings: partial pressure of oxygen, 60.3 Torr; partial pressure of carbon dioxide, 30.4 Torr; and pH, 7.446. A blood analysis revealed the following findings: white blood cell count, 41 900/μL (neutrophil, 88.0%; lymphocytes, 5.0%; monocytes, 2.0%; promyelocytes, 1.0%; and myelocytes, 3.0%; metacytes, 1.0%); hemoglobin, 7.8 g/dL; platelet count, 34.2 × 10^4^/μL; lactate dehydrogenase, 564 IU/L, Krebs von den Lugen‐6, 2826 U/mL; and carcinoembryonic antigen, 15.6 ng/mL. The patient was negative for β‐D glucan and cytomegalovirus antigen. HRCT showed diffuse GGOs in both lung fields (Figure 1C,D).

On the following day, we performed bronchoscopy with bronchoalveolar lavage in the right middle lobe. The bronchoalveolar lavage fluid showed a light milky appearance (Figure 2B) and was periodic acid‐Schiff (PAS)‐positive. Transbronchial lung biopsy revealed the precipitation of dense, homogenous, eosinophilic material, which had a fine granular appearance, and which filled the alveoli (Figure 2C); however, no evidence of OP was found. Based on these findings, he was diagnosed with PAP. Although the granulocyte/macrophage colony‐stimulating factor autoantibody level was not measured, the diagnosis of sPAP was confirmed by compatible radiological findings and a pre‐existing diagnosis of aCML.^3^, ^4^

He gradually developed respiratory failure with the progression of PAP; whole‐lung lavage was subsequently performed. Unfortunately, the patient died of acute enteritis within 1 month of whole‐lung lavage.

## 3 DISCUSSION

Our patient with aCML developed OP, and we started corticosteroid treatment. Despite initial improvement, his condition deteriorated due to progression of PAP and he ultimately died of acute enteritis.

In the present case, the patient developed both OP and PAP during the course of aCML. A previous study reported that OP is a rare diagnosis with an estimated incidence of 34 per 100 000 patients with hematologic malignancy.^5^ PAP is also a rare disorder that predominantly affects the lungs. PAP is clinically classified into three distinct forms: autoimmune PAP, sPAP, and congenital PAP. sPAP results from underlying diseases that presumably impair surfactant clearance due to abnormalities in the number and function of alveolar macrophages. Ishii et al^3^ reported that hematologic malignancies were the most common form of underlying disease, with 88% of cases involving hematological disorders. Cordonnier et al^6^ reported that sPAP was present as a complication in 5.5% of patients with hematologic disorders. To our knowledge, there have been no reports in which both OP and PAP developed as complications of hematologic disorders in the same patient. The precise mechanisms of complication of both OP and PAP in our patient remain unclear. Several factors are hypothesized to be involved in the relationship between hematologic disorders and pulmonary involvement, such as the production of superoxide anions by the neutrophils, immunological abnormalities, genetic factors, and chromosomal abnormality^7^, ^8^, ^9^, ^10^; however, further studies will be needed to clarify this issue.

In our patient, chest CT initially showed nodular and patchy lesions indicative of OP, followed by diffuse GGO indicative of sPAP. The typical radiological pattern of sPAP is GGO with or without septal thickening,^4^, ^11^ with diffuse infiltration common in OP.^12^ Autoimmune PAP has also been reported to be associated with myeloproliferative neoplasms.^13^ Although the GM‐CSF autoantibody level was not measured in our patient, the diagnosis of sPAP was confirmed by these compatible radiological findings.^4^ GGO and septal thickening are nonspecific findings in patients with hematological malignancies, and we misdiagnosed the patient with a relapse of OP and increased his steroid dosage. It is important that physicians include sPAP in the differential diagnosis when they encounter patients with pre‐existing hematological diseases who show new pulmonary opacities on chest radiography or CT. In our patient, bronchoalveolar lavage and a transbronchial lung biopsy were diagnostically useful, and these tests are recommended for obtaining an accurate diagnosis; however, such procedures may sometimes be difficult to perform in patients with severe conditions.

OP complicated with hematologic malignancies responds favorably to corticosteroid therapy.^5^ Indeed, our patient initially improved on steroid treatment; however, PAP became apparent during treatment and resulted in a fatal outcome. The prognosis of patients with hematologic disorders complicated by sPAP is poor (2‐year survival rate, 46%; median survival time 16 months).^3^ Whole‐lung lavage, which is the gold‐standard therapy for autoimmune PAP,^14^, ^15^ has been reported to be ineffective against sPAP.^16^ Furthermore, corticosteroid treatment was reported to be associated with poor survival in patients with sPAP.^16^ Steroids are known to both increase the production of phospholipids and depress the monocyte function,^6^ which may be associated with the development and worsening of PAP. Our patient, who was being treated with corticosteroids for OP, died 2 months after the diagnosis of PAP, and steroid therapy may have been associated with his poor outcome. Although OP often responds well to steroid therapy,^5^ the indications of steroid therapy for OP complicated with hematologic malignancies remain unclear. Although no obvious findings of PAP were detected in the first BAL fluid and transbronchial lung biopsy specimens, PAP may have already developed at the first bronchoscopy and may have been worsened by steroid treatment. A delay in the diagnosis can lead to respiratory failure in PAP, thus leading to a situation in which whole‐lung lavage can be challenging to perform.^17^ Physicians should be alert for the possibility of developing or worsening PAP complicated with OP, as the steroid therapy used for OP has an unfavorable effect on the clinical course of such patients.

In conclusion, we encountered a case of PAP that developed during steroid treatment for OP in a patient with a hematologic malignancy. Physicians should be alert for this possibility when patients with hematologic malignancies develop lung complications, including PAP and OP. The indications of corticosteroid therapy in patients with hematologic malignancy‐associated OP should be discussed in the future.

## CONFLICT OF INTEREST

The authors declare no conflicts of interest in association with the present study.

## AUTHOR CONTRIBUTION

CH: is the guarantor of the paper, taking responsibility for the integrity of the work as a whole, from inception to published article. KS, SF, YY, NW, HM, YK, YS, AK, YE, and KK: aggregated the data, created the figures, and helped draft the discussion of the manuscript.
